# Effects of Dietary Protein Levels on the Growth, Physiological, and Biochemical Indices of Juvenile Yellow River Carp (*Cyprinus carpio haematopterus*)

**DOI:** 10.3390/ani15121800

**Published:** 2025-06-18

**Authors:** Xiaona Jiang, Feihu Qu, Yanlong Ge, Chitao Li, Xiaodan Shi, Xuesong Hu, Lei Cheng, Xinyu Zhao, Zhiying Jia

**Affiliations:** 1 Heilongjiang River Fisheries Research Institute, Chinese Academy of Fishery Sciences, Harbin 150076, China; jiangxiaona@hrfri.ac.cn (X.J.); qufeihu@hrfri.ac.cn (F.Q.); geyanlong@hrfri.ac.cn (Y.G.); lichitao@hrfri.ac.cn (C.L.); shixiaodan@hrfri.ac.cn (X.S.); huxuesong@hrfri.ac.cn (X.H.); chenglei@hrfri.ac.cn (L.C.); zhaoxinyu@hrfri.ac.cn (X.Z.); 2 Key Laboratory of Freshwater Aquatic Biotechnology and Breeding, Ministry of Agriculture and Rural Affairs, Harbin 150076, China

**Keywords:** Yellow River carp, dietary protein, regression equations, muscle quality, enzyme activities

## Abstract

An appropriate protein content in the diet can effectively increase the growth rate, muscle quality, and environmental stress resistance of fish. In this study, juvenile Yellow River carp (51.56 ± 0.17 g) were fed isofat diets with different protein concentrations (22%, 25%, 28%, 31%, 34%, and 37%). The results showed that, compared with other protein content groups, when the protein content was 34%, the WGR, SGR, and FCR were significantly higher, while the FCR was significantly lower (*p* < 0.05). Among them, the SGR and FCR were positively correlated with the dietary protein content, with the regression equations being y_1_ = −32.208x^2^ + 21.897x − 1.4001 (R^2^ = 0.8622) and y_2_ = 97.027x^2^ − 68.428x + 13.269 (R^2^ = 0.9663), respectively. When x was 33.99% and 35.26%, the SGR and FCR had extreme values. The contents of CP, Lys, EAA, and EAA/TAA were significantly greater in the 34% protein group, reflecting muscle quality (*p* < 0.05). In addition, the activities of α-AMS, LPS, TPS, SOD, CAT, and GSH-Px were significantly greater in the 34% protein group (*p* < 0.05). Similarly, the relative expression levels of *GH*, *IGF-I*, *TOR*, *4EBP2*, *Rhag*, *Rhbg*, and *Rhcg1* were significantly greater in the 34% protein group compared to the other protein groups (*p* < 0.05). The above results indicated that when the protein content in the diet was 34%, it significantly improved the growth and stress resistance of juvenile Yellow River carp. Based on the nonlinear regression equations for the SGR and FCR, the optimal dietary protein content of juvenile Yellow River carp (51.56 ± 0.17 g) was determined to be 33.99–35.26%.

## Simple Summary

Dietary protein in the diet is a crucial nutrient for the immune system and growth of fish, which is also the most expensive component in the carp feed industry. Furthermore, a high protein content in the daily diet can promote deterioration of the water environment. The Yellow River carp (*Cyprinus carpio haematopterus*) is widely farmed due to its advantages, such as delicious meat, strong disease resistance, and fast growth rate. In this study, by feeding isofat feeds with different protein levels (22%, 25%, 28%, 31%, 34%, and 37%) and a fat content of approximately 7%, the growth performance, serum biochemical indicators, muscle quality, activities of digestive and antioxidant enzymes, and relative expression of functional genes associated with different protein contents were determined. The results indicated that the optimal dietary protein content for juvenile Yellow River carp (51.56 ± 0.17 g) is 33.99–35.26%. This study could promote the sustainable development of aquaculture from the perspectives of nutrition, the economy, and the environment.

## 1. Introduction

Protein is a vital nutrient for the immune system and growth of fish. The protein requirement of fish refers to the minimum protein intake that satisfies their maximum growth requirements. When the feed protein content is too low, the fish cannot reach their optimal growth rate, affecting their muscle quality [1]. However, when the dietary protein content is too high, it can lead to metabolic disorders in fish, which is not conducive to their healthy growth. Furthermore, it can also lead to high nitrogen content in excreta, promoting deterioration of the water environment [2,3]. More importantly, as a vital core nutrient, protein is also the most expensive ingredient in the carp feed industry [4]. The expression of different genes in fish is closely related to their growth and health. For example, the expression of *GH*, *IGF-I*, *TOR*, and *4EBP1* can regulate the synthesis of proteins in fish and affect their growth [5], while *Rhag*, *Rhbg*, and *Rhcg1* are marker factors of ammonia metabolism in fish [6]. Due to its high protein content, good palatability, easy absorption, and balanced composition of essential amino acids, fish meal has become a high-quality protein source in the aquaculture industry. At present, the global fishmeal supply is tight and is insufficient to meet the associated demand; thus, determining the optimal protein requirements of fish can promote the sustainable development of aquaculture from the perspectives of nutrition, the economy, and the environment.

The common carp (*Cyprinus carpio*) is the third most widely farmed freshwater fish in the world due to its wide range of suitable environmental temperatures, low nutritional requirements, and strong stress resistance [7]. The Yellow River carp (*Cyprinus carpio haematopterus*) is widely cultivated due to its delicious meat, strong disease resistance, and fast growth rate [8]. Previous studies have shown that protein levels in feed are related to fish body size and generally decrease with increasing fish body weight [9]. The optimum dietary protein level for the Yellow River carp (160 ± 15.56 g) is 25–28%, which results in the fastest growth rate and high nutritional value of muscle [10]. Currently, only dietary supplement supplementation has been reported to significantly affect the growth performance and fillet texture of the juvenile Yellow River carp [8,11], and there have been few studies on the optimal dietary protein level for the fish. In this study, we determined the effects of different dietary protein contents on growth performance, muscle quality, serum biochemical indices, antioxidant oxidase, and digestive enzyme activity activities, and the expression of functional genes in juvenile Yellow River carp (51.56 ± 0.17 g), aiming to determine the optimal dietary protein content of these fish (51.56 ± 0.17 g) and provide data supporting the breeding of juvenile carp with low dietary protein requirements.

## 2. Materials and Methods

### 2.1. Experimental Material and Diets

The juvenile Yellow River carp (51.56 ± 0.17 g) used in this study were obtained from the Kuandian Fisheries Experiment Station of the Heilongjiang River Fisheries Research Institute, Chinese Academy of Fishery Sciences. We designed the feed formula based on a common carp feed formula [12]. The daily diet mainly consisted of fish meal and casein as the main protein sources and fish oil and soybean oil as the main fat sources. Six different diets with protein contents of 22%, 25%, 28%, 31%, 34%, and 37% and similar crude lipid contents were fed to the juvenile Yellow River carp (Table 1). Microcrystalline cellulose was used to regulate the protein gradients in the diets. All solid substances (i.e., excluding fish oil, soybean oil, and water) were ground into powder and mixed together in a predetermined proportion. Then, the liquid was added to make granules with a diameter of 2 mm using the laboratory pellet machine. After the particles were dried at 60 °C for 5 h, they were packed into plastic-lined bags and stored in a refrigerator at −20 °C. The feed components in this study were all tested by Qingdao Stand Testing Co., Ltd. (Qingdao, China).

### 2.2. Experimental Design and Feeding Management

The juvenile Yellow River carp were raised temporarily for two weeks before the experiment and fed commercial feed (Tong Wei Co., Ltd., Chengdu, China, model:101) to fully adapt to the feeding environment. A total of 216 healthy and disease-free juvenile Yellow River carp were randomly placed in 18 aquariums (46 cm × 32 cm × 52 cm) with 6 experimental groups and 3 replicates per group. Before the formal test began, the fish were starved for 24 h. Each feeding amount was 3% of body weight, and the water in the tank was changed three times per day [13]. The water temperature of the test was controlled at 25 ± 1 °C, the dissolved oxygen concentration in the water was 6–8 mg/L, the pH was controlled at 6.5~7.5, and the ammonia nitrogen concentration was ≤0.02 mg/L during the test. The temperature, dissolved oxygen concentration, pH, and ammonia nitrogen concentration were controlled using the temperature controller in the circulation system and the water quality detection equipment instrument. The experimental period was maintained for 8 weeks.

### 2.3. Sample Collection and Fish Performance

Before collection, the feeding was stopped for 24 h. All the experimental fish were anesthetized with fish anesthetic (MS-222, 100 mg/L; Beijing Green Hengxing Biological Technology Co., Beijing, China). The weights of the 12 carp in each experimental group were measured, the amount of feed fed in each tank and the number of live fish in each group were counted, and 9 carp were selected for tissue collection. The blood was collected from the tail vein of the experimental fish and placed in a centrifuge tube. Then, they were stored at 4 °C for 1–2 h and centrifuged at 3500 r/min for 10 min. Next, they were left to stand at 4 °C for 1–2 h and centrifuged at 3500 r/min for 10 min. We extracted the upper layer of serum, loaded it into centrifuge tubes, and stored it at −20 °C until the serum biochemical indices were determined. The muscle, liver, intestinal, brain, and gill tissues of the experimental fish were placed in liquid nitrogen and then stored in a −80 °C freezer. In addition, the SR was calculated according to the difference between the initial and final number of individuals, and the WGR and SGR were calculated according to the difference between the initial and final number of individuals. The FCR and PER were calculated based on body weight and feed amount, and the formulas used were as follows:

(1) $$\mathrm{SR}(\%)=N_{t}/N_{0}\times 100\%$$

(2) $$WGR(\%)=(W_{t}-W_{0})/W_{0}\times 100\%$$

(3) $$SGR(\%/d)=\left[(\ln W_{t}-\ln W_{0})/t\right]\times 100\%$$

(4) $$\mathrm{AGR}\;(g/\mathrm{fish}/d)=(W_{\mathrm{et}}-W_{e0})/t$$

AF (g/d) = F_e_/t (5)

(6) PI(g/d)=AF×P

(7) $$PER(\%)=\left[\left(W_{t}-W_{0}\right)/\left(F\times P\right)\right]\times 100\%$$

(8) $$FCR=\left[F/(W_{t}-W_{0})\right]$$

(9) $$\mathrm{FER}=\left[(W_{t}-W_{0})/F\right]$$

where N_t_ is the survival number of surviving fish, N_0_ is the initial number of fish, W_t_ is the final body weight (g), W_0_ is the initial body weight (g), F is the food intake (g), t is the breeding period (d), W_e0_ is the initial body weight of each fish, W_et_ is the final body weight of each fish, F_e_ is the food intake of each fish, and P is the feed crude protein content (%).

### 2.4. Index Measurement

The approximate composition of the test fishball feed and muscle was evaluated according to AOAC (2005) [14] standard procedures. The muscle moisture content of the experimental fish was obtained by using a vacuum freeze dryer (FD-1A-50, Yuming, Beijing, China). The contents of CP and CL in the muscle of the experimental fish were determined using the Kjeldahl nitrogen determination method (GB 5009.5-2016) [15] and the Soxhlet extraction method (GB5009.6-2016) [16], respectively. The crude ash content was determined by burning the sample to a constant weight at 550 °C. The amino acid composition of the muscle was determined through chromatography (1260 and 7890 A, Agilent, Santa Clara, CA, USA). The contents of amino acids and fatty acids were determined, respectively, with the acid hydrolysis method (GB5009.124) [17] and gas chromatography/mass spectrometry. After static grinding, the intestinal and liver tissues were mixed with normal saline (1:9) at a low temperature to detect the intestinal lipase activity and liver antioxidant indices via an enzyme activity detection kit (Jiancheng, Nanjing, China). The liver and intestinal enzyme activity indicators, including TP (A045-2, Coomassie brilliant blue method), GSH-Px (A005-1, colorimetric method), CAT (A007-1-1, ammonium molybdate method), MDA (a001-1-1, TBA), SOD (A001-3, WST-1 method), α-AMS (A045-2, starch-to-iodine colorimetric method), LPS (A054-2-1, microplate method), and TPS (A080-2, quartz colorimetry), were measured. Serum biochemical indices were determined via immunoturbidimetry using TP (105-000451-00), ALP (105-000476-00), and UA (105-000444-00) kits purchased from Mindray Corporation (Shenzhen, China). All indices were determined with a biochemical analyzer (BS350E, Mindray, Shenzhen, China). All the trials measured 9 individuals per group.

### 2.5. RNA Extraction and Quantitative Real-Time PCR (qRT–PCR)

Total RNA was extracted from the brain, intestinal, and gill tissues of the experimental fish via the RNeasy Mini Kit (Qiagen, Hilden, Germany). According to the instructions of the RNeasy Mini Kit, the extracted RNA was purified. The concentration and quality of RNA were detected [13]. The PrimeScript™Reagent Kit with gDNA Eraser (TaKaRa, Beijing, China) was used to reverse total RNA. All specific primers in this study were designed by Primer Premier 5.0 (Supplementary Table S1). qRT-PCR was performed by using the ABI7500 system (Life Technologies, Carlsbad, CA, USA) according to the TB Green™Premix Ex Taq™II (TaKaRa, Beijing, China) instructions. The specificity of the primers was confirmed by analyzing the melting curve. *β-actin* was used as the internal reference gene, which has been reported to be the most suitable reference gene in mirror carp [18,19], and its expression remained highly stable across the samples. For the negative control, double distilled water was used instead of a template. The reaction procedure is as follows: pre-denaturation at 95 °C, 30 s; PCR reaction: 95 °C, 5 s; Tm of different primers, 34 s, 40 cycles; melting curve: 95 °C, 15 s; 60 °C, 1 min; 95 °C, 15 s. The mRNA expression of seven genes was calculated using the 2^(−ΔΔCt)^ method. There were at least three replicates per trial group. The repeated values of Tm comprised three replicates per trial group. The qRT-PCR consisted of 3 biological replicates and 4 technical replicates.

### 2.6. Data Analysis

In this study, the differences in the trial parameters among the fish fed different test diets were analyzed via one-way analysis of variance (ANOVA). Multiple comparisons utilizing Duncan’s test were performed on the variables if significant differences were detected. All data are presented as the mean values ± SDs of at least three replicates. Analysis of the experimental data was performed using the IBM SPSS software (version 22.0, IBM Corp., Armonk, NY, USA). Nonlinear regression analysis was performed to analyze the relationships between SGR, FCR, and dietary protein content. All of the data were checked for a normal distribution with a one-sample Kolmogorov–Smirnov test, and homogeneity of variances was assessed via Levene’s test. This study repeated all the experiments at least three times.

## 3. Results

### 3.1. Growth Performance

**Figure 1:**
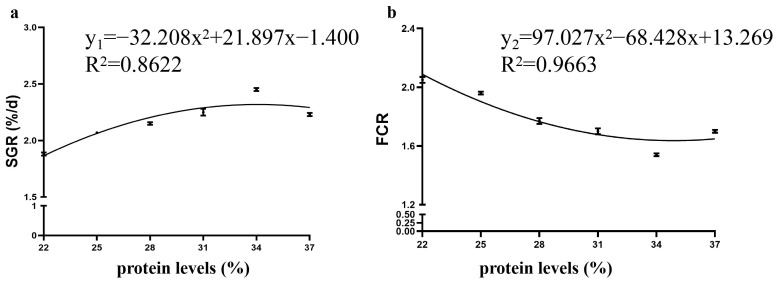
Relationships between the SGR (y_1_), FCR (y_2_), and dietary protein level (x) of the Yellow River carp (*Cyprinus carpio haematopterus*). The regression equations were y_1_ = −32.208x^2^ + 21.897x − 1.4001 (R^2^ = 0.8622, *p* < 0.05) and y_2_ = 97.027x^2^−68.428x + 13.269 (R^2^ = 0.9663, *p* < 0.05). (**a**) SGR; (**b**) FCR.

The results are shown in Table 2. As the dietary protein content increased, FBW, WGR, SGR, and PER first increased but then decreased. The highest values were found in the 34% protein group, whereas the lowest values were found in the 22% protein group (*p* < 0.05). The AGR, AF, and PI gradually increased with an increase in protein content in the daily diet. The FER value was the highest in the 34% protein group, significantly higher than that in other protein groups (except the 31% protein). With SGR and FCR as the dependent variables y_1_ and y_2_ and the feed protein content as the independent variable x, a fitting curve analysis was subsequently carried out (Figure 1). The fitted regression equations obtained were y_1_ = −32.208x^2^ + 21.897x − 1.4001 and y_2_ =97.027x^2^ − 68.428x + 13.269. When x = 33.99%, the SGR value was maximal (Figure 1a), and when x = 35.26%, the FCR value was minimal (Figure 1b).

### 3.2. Basic Nutrients

The results revealed that with increasing dietary protein content, the CP content first increased but then decreased, and the CL content gradually decreased (Table 3). The CP content in the 34% protein content group was significantly higher than that in the 22 and 25% protein groups (*p* < 0.05). The CL content in the 34% protein group was significantly lower than that in the 22% protein group (*p* < 0.05). With increasing dietary protein content, the contents of Thr, Val, Ile, Lys, EAA, FAA, TAA, and EAA/TAA first increased but then decreased, whereas the contents of Glu and Cys gradually increased (Table 3). Compared with those in the other protein groups, the Lys, EAA, TAA, and EAA/TAA contents were significantly greater in the 34% protein group, whereas the Lys, EAA, and TAA contents in the 22% and 25% protein groups were significantly lower (*p* < 0.05). The contents of Glu and Cys in the 22% and 25% protein content groups were significantly lower than those in the 34% and 37% protein groups (*p* < 0.05). In addition, the NEAA/TAA and HEAA/TAA values decreased with increasing dietary protein content, and the values in the 37% protein content group were significantly lower than those in the other protein content groups except for the 34% protein group (*p* < 0.05). Furthermore, the amino acid contents of C14:0, SFA, PUFA, DHA+EPA, and n-6 PUFA gradually increased with increasing dietary protein content (Table 4). The contents of PUEA and n-6 PUEA in the muscle of the 22% protein group were significantly lower than those in the other protein groups (*p* < 0.05). The DHA+EPA content in the 22% protein group was significantly lower than those in the 31, 34 and 37% protein groups (*p* < 0.05).

### 3.3. Serum Biochemical Indices

The contents of TP, ALP, and UA in the serum of the Yellow River carp in different dietary protein groups were determined. The results revealed that with increasing dietary protein levels, the serum ALP content first increased but then decreased, and the serum UA content increased gradually (Table 5). The ALP contents in the 31%, 34%, and 37% protein contents groups were significantly higher than those in the other content groups, whereas the UA contents in the 22 and 25% protein groups were significantly lower (*p* < 0.05). There was no significant change in the TP content among the six protein content groups.

### 3.4. Digestive Enzyme Activity

**Figure 2:**
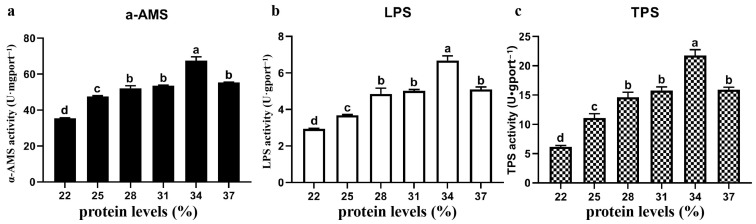
The effects of different protein levels in the feed on the digestive ability of the Yellow River carp. The activities of the digestive enzymes (**a**) α-AMS (U/mgport^−1^), (**b**) LPS (U/gprot^−1^), and (**c**) TPS (U/gprot^−1^) were assayed in the intestine. Significant differences among groups are represented by different letters (*p* < 0.05).

The activities of digestive enzymes in intestinal tissues enriched with different protein contents were analyzed. As shown in Figure 2, with increasing dietary protein content, the enzyme activities of α-AMS, LPS, and TPS presented similar trends, all of which first increased but then decreased, with the highest value occurring in the 34% protein group and the lowest value occurring in the 22% protein group (*p* < 0.05). In addition, except for the 34% protein content group, the enzyme activities of α-AMS, LPS, and TPS in the 28, 31, and 37% protein content groups were significantly higher than those in the other groups (*p* < 0.05).

### 3.5. Antioxidant Oxidase Content

**Figure 3:**
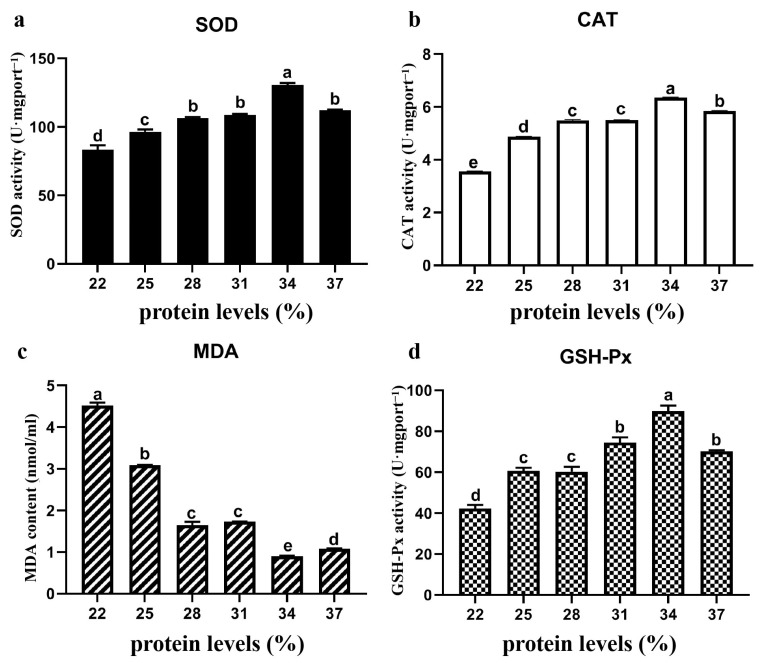
The influence of different protein contents in the diet on the antioxidant capacity of the Yellow River carp. The levels of antioxidant oxidases ((**a**) SOD (U/mgprot^−1^); (**b**) CAT (U/mgprot^−1^); (**c**) GSH (umol/gprot^−1^); (**d**) MDA (nmol/mL)) in the liver tissues of different protein content groups were assayed. Significant differences among groups represented by different letters (*p* < 0.05).

The activities of antioxidant oxidase in liver tissues enriched with different protein contents were determined, and the results are shown in Figure 3. The results revealed that the activities of the SOD, CAT, and GSH-Px enzymes first increased but then decreased with increasing dietary protein content, whereas the MDA content showed the opposite trend. Compared with those in the other protein groups, the SOD, CAT, and GSH-Px activities in the 34% protein group were significantly greater, whereas the MDA content in the 34% protein group was the lowest (*p* < 0.05). In the 22% protein content group, the MDA content was significantly higher, and the SOD, CAT and GSH-Px activities were significantly lower (*p* < 0.05).

### 3.6. Expression of Genes Related to Growth and Protein Synthesis

**Figure 4:**
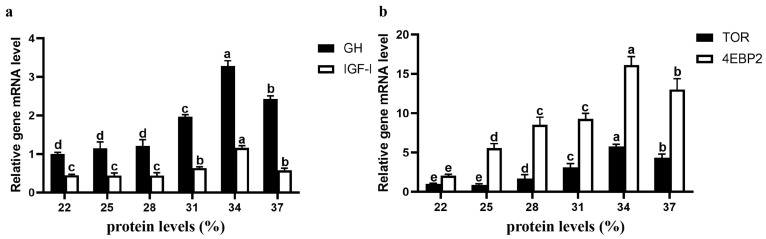
The effects of dietary protein content on the mRNA expression levels of *GH* and *IGF-I* in brain tissue and *TOR* and *4EBP2* in liver tissue, respectively. The mRNA expression level of the GH (**a**) or TOR (**b**) at 22% protein content was considered to be 1. (**a**) *GH* and *IGF-I*; (**b**) *TOR* and *4EBP2*. Lowercase letters indicate that the relative gene expression levels of the same gene significantly differ between protein content groups (*p* < 0.05).

With increasing dietary protein content, the relative expression levels of the *GH*, *IGF-I*, *TOR*, and *4EBP2* first increased but then decreased (Figure 4). The relative expression levels of *GH*, *IGF-I*, *TOR*, and *4EBP2* in the 34% protein group were significantly higher than those in the other protein groups (*p* < 0.05). Except for those in the 34% protein group, the relative expression levels of *GH* and *IGF-I* were significantly higher in the 31 and 37% protein groups, and the mRNA expression levels of *TOR* and *4EBP2* were significantly greater in the 37% protein group than in the other protein groups (*p* < 0.05). In addition, the mRNA expression levels of *TOR* and *4EBP2* in the 22% and 25% protein content groups were significantly lower than those in the other protein content groups (*p* < 0.05).

### 3.7. Expression of Genes Related to Metabolism

**Figure 5:**
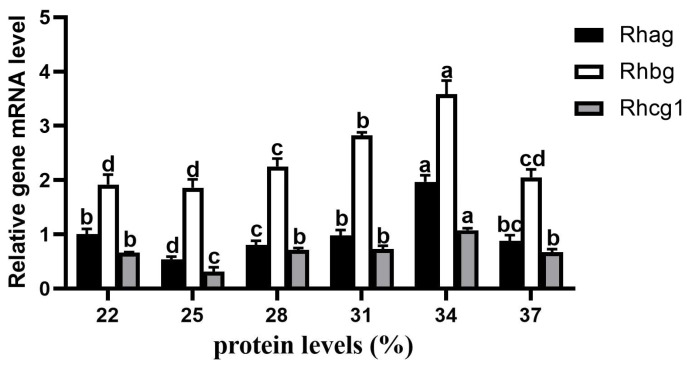
Effect of dietary protein levels on *Rhag, Rhbg*, and *Rhcg1* mRNA expression in the Yellow River carp. The mRNA expression level of the *Rhag* at 22% protein content was considered to be 1. Lowercase letters indicated that the relative gene expression levels of the same gene significantly differ between protein content groups (*p* < 0.05).

The relative expression levels of *Rhag*, *Rhbg*, and *Rhcg1* in gill tissues from different protein groups were detected using qRT-PCR (Figure 5). With increasing dietary protein content, the relative expression levels of *Rhag* and *Rhcg1* first decreased, then increased, and finally decreased, whereas the *Rhbg* mRNA expression first increased but then decreased. Similarly, the *Rhag*, *Rhbg*, and *Rhcg1* mRNA expression levels in the 34% protein group were significantly higher than those in the other protein groups (*p* < 0.05). In addition, the relative expression levels of *Rhag* and *Rhcg1* in the 22% protein group were significantly higher than those in the 25% protein group (*p* < 0.05).

## 4. Discussion

### 4.1. Growth

Protein is the most essential nutrient for fish, as the growth of the fish body essentially involves the accumulation of protein. Therefore, a proper protein content in the diet is beneficial for the growth and development of fish. A protein content that is too high or too low may lead to arrested fish growth, low immunity, metabolic dysfunction, and other detrimental effects. In this study, with increasing dietary protein content, the WGR and SGR first tended to increase but then decreased, and the values of both in the 34% protein group were significantly higher than those in the other protein groups (*p* < 0.05). The results indicated that dietary protein content could significantly affect the growth rate of the Yellow River carp and that too much or too little dietary protein could reduce the growth rate. These results are similar to those reported for the Nile tilapia (*Oreochromis niloticus*) (Linnaeus, 1758) [1], the grouper (*Epinephelus malabaricus*) (Bloch and Schneider, 1804) [20], and the orange-spotted grouper (*Epinephelus coioides*) (Hamilton, 1822) [21]. PER and FCR can be used as important indicators to measure the relationship between weight gain and the amount of food consumed by fish during the breeding process. PER reflects the actual effect of fish utilization of food, whereas FCR has the opposite effect. This study revealed that with increasing feed protein content, the trends of PER and FCR in juvenile fish tended to be opposite, reaching significant extremes at 34% protein. The results revealed that the juvenile Yellow River carp (51.56 ± 0.17 g) had a better utilization rate when the dietary protein content was 34% and had the best growth performance when the dietary protein content was 33.99–35.26%. Studies have shown that when the feed protein content of carp (2.20 ± 0.003 g) is 38%, a significantly higher SGR and lower FCR can be observed [22]. Previous studies have shown that the dietary protein content of juvenile common carp (10.00 ± 1.15 g) for optimal growth performance is 31–32% [23] and that a dietary protein content of 30–32% can satisfy the requirements of normal growth and a high feed utilization rate in common carp (75.38 ± 0.18 g) [24]. Furthermore, research has revealed that the optimal feed protein requirement for Yellow River carp (160.24 ± 15.56 g) is 25–28% [10]. The above results indicate the optimal dietary protein content requirements for common carp of different weights, and based on these results, we speculated that the dietary protein requirements of common carp might be inversely proportional to the size of the fish. The change in water temperature also affects the food intake of common carp [25]. Using fish meal as the protein source and with a water temperature of 28–31 °C, the optimal feed protein requirement for the common carp (13.12 ± 0.55 g) was shown to be 27.60% [26]. When the water temperature was 19–22 °C, the optimal feed protein requirement for the common carp (10 ± 1.2 g) was 31% [27]. In this study, the water temperature for the juvenile Yellow River carp (51.56 ± 0.17 g) was 24–26 °C, and the optimal protein in the feed was 33.99–35.26%. It can be speculated that the dietary protein requirements of the common carp might vary due to different experimental water temperatures, specifications, protein sources, and other reasons.

### 4.2. Muscle Quality

The nutritional components of fish muscle are important factors affecting the ultimate nutritional value of the fish [28]. Dietary protein in the diet plays an important role in synthesizing EAA and maintaining the protein balance in fish [29]. In this study, the CP content was significantly greater in the 34% protein group, whereas it was significantly lower in the 22% and 25% protein groups (*p* < 0.05), indicating an appropriate dietary protein content can significantly increase the CP content in the muscle. It is speculated that the ingested protein is digested to repair and update the protein tissue in the fish, thereby leading to an increase in the protein content of the entire fish muscle. As the protein content in the diet gradually increased, the CL content tended to decrease, which is similar to the results of previous studies [21,30]. In addition, the CL content in the 22% protein group was significantly greater than that in the 34% protein group (*p* < 0.05), suggesting that low-protein diet feeding led to insufficient protein synthesis in the juvenile Yellow River carp, affecting lipid metabolism and resulting in lipid deposition in the muscle. It is reasonable to speculate that when the protein content in the daily diet increases to a level that satisfies a fish’s needs, it promotes the synthesis of lipid metabolism enzymes in the body, thereby inhibiting lipid deposition. The contents of amino acids and fatty acids in muscle can serve as the key indicators for evaluating the nutritional value and flavor of fish meat [31]. At present, EAAs play an important role in maintaining human health, among which Lys has antioxidant and anticancer effects [32]. The contents of Lys, EAA, TAA, and EAA/TAA in the muscle of the juvenile Yellow River carp were significantly greater in the 34% protein group (*p* < 0.05), indicating that feeding the juvenile Yellow River carp with a protein content of 34% resulted in greater nutritional value of the muscle. The contents of PUFA and n-6 PUEA—the main source of unsaturated fatty acids for human intake—in the muscle of the juvenile Yellow River carp were significantly lower in the 22% protein group than in the 31, 34, and 37% protein groups. In addition, the contents of DHA+EPA in the 22% protein group were significantly lower than those in the 31, 34, and 37% protein groups (*p* < 0.05). These results suggest that the protein content in the diet can significantly affect the content of unsaturated fatty acids in the muscle of the juvenile Yellow River carp.

### 4.3. Biochemical Indicators

Biochemical indicators in serum can be used to assess the physiological metabolic status, health condition, and adaptability of fish to the environment [33]. ALP in the serum of vertebrates plays an important role in phosphate hydrolysis and membrane transport [21]. In serum, changes in UA levels can not only reflect metabolic and immune capabilities but may also be related to the body’s waste excretion ability [34]. In this study, the ALP content was significantly highest in the 34% protein group and significantly lowest in the 22% protein group (*p* < 0.05), indicating that when the feed protein content of the Yellow River carp was 34%, the increase in ALP content in the serum of the common carp might promote phosphate hydrolysis and membrane transport, which could be related to growth. The UA contents in the 22% and 25% protein content groups were significantly lower than those in the other protein content groups (*p* < 0.05), suggesting that as the daily dietary protein content gradually increased, the nitrogen metabolism in the Yellow River carp was blocked, preventing the normal excretion of harmful toxins and resulting in an increase in UA in the serum. This finding is consistent with the results obtained in Yellow River carp (160.24 ± 15.56 g) when considering a similar experimental period [10].

### 4.4. Physiological Indicators

Enzymes are important catalysts in living organisms that play a significant role in their growth and immunity. Studies have shown that the activities of digestive enzymes can determine the digestive and absorptive capacities of fish [35]. The enzyme activities of α-AMS, LPS, and TPS in the intestinal tissue of the juvenile Yellow River carp gradually increased in fish fed with 22% to 34% dietary protein content and were significantly greater in the 34% protein content group than those in the other protein content groups (*p* < 0.05). When the protein content in the diet exceeded 34%, the activities of the three digestive enzymes began to decrease. It is speculated that excessive protein content in the feed can inhibit the digestive enzyme activities in the juvenile Yellow River carp, which is consistent with previous research results [21]. SOD, CAT, and GSH-Px are important antioxidant enzymes for maintaining the balance of oxidation and antioxidation in the body and play important roles in maintaining the survival of cells in fish and preventing damage to the body [36]; their main function is to eliminate unnecessary free radicals in the organism and prevent excessive oxidation of the body. However, an increase in the MDA content is regarded as a definite indicator of lipid peroxidation [37]. With increasing dietary protein content, the enzymatic activities of SOD, CAT, and GSH-Px in the liver of the juvenile Yellow River carp first increased but then decreased, and all were significantly greater in the 34% protein group. The content of MDA first decreased but then increased and was significantly lower in the 34% protein group (*p* < 0.05). These results indicate that when the dietary protein content was 34%, the liver tissue of the juvenile Yellow River carp had a strong antioxidant capacity. Combining the results for digestive enzymes and antioxidant enzymes, it is speculated that both insufficient and excessive intake of dietary protein could reduce the digestive capacity and antioxidant capacity of the juvenile Yellow River carp, thus affecting their growth and immune performance. Similar results have been reported in studies of the juvenile common carp [38], the large yellow croaker (*Aristichthys nobilis*) (Richardson) [39], the juvenile small yellow croaker (*Larimichthys polyactis*) (Perciformes: Sciaenidae) [40], and the Chinese rice field eel (*Monopterus albus*) [41], all considering experimental periods of 7 to 8 weeks.

### 4.5. Gene Expression

Genes are involved in a variety of important biological functions, such as growth, immunity, and the maintenance of cellular and collective homeostasis. In organisms, *GH* and *IGF-I* are important factors affecting protein synthesis and storage. The expression of *IGF-I* is also regulated by *GH*, which can control the growth and protein synthesis of fish [4]. Many studies have shown that the expression of *GH* is also related to dietary differences [42,43]. Protein deposition is an important factor for weight gain in fish [9,44]. *TOR* and *4EBP2* are involved in protein synthesis in the muscles and intestines of fish [6]. *TOR* can promote the initiation of transcription and protein expression by directly phosphorylating *4EBP2*, thereby facilitating protein synthesis in the body. This study revealed that with increasing feed protein content, the relative expression levels of *GH*, *IGF-I*, *TOR*, and *4EBP2* in the brain tissues of the juvenile Yellow River carp all tended to first increase but then decreased and were significantly greater in the 34% protein content group than those in the other protein content groups (*p* < 0.05). The high relative expression of *GH* and *IGF-I*, as well as *TOR* and *4EBP2*, may promote protein synthesis in muscles; this might also be one of the reasons why the juvenile Yellow River carp had the largest WGR and SGR in the 34% protein group and the highest contents of crude protein and EAA, suggesting that feeding too little or too much protein can inhibit muscle growth and protein synthesis in the carp. Similarly, consistent results have also been reported in the juvenile giant grouper (*Epinephelus lanceolatus*) [45].

Key metabolic processes such as osmotic pressure, ion balance, and ammonia nitrogen excretion are regulated within the fish body through the epidermal cells of the gill filaments [46,47]. Excessive protein metabolism in fish requires a large amount of energy, which might be an important reason why a diet with a high protein content inhibits fish growth [48]. At present, Rh family genes such as *Rhag*, *Rhbg*, and *Rhcg1* are generally regarded as direct participants in the active emission of ammonia in gills [49,50]. The results of this study revealed that the mRNA expression levels of *Rhag*, *Rhbg*, and *Rhcg1* gradually increased in the gill tissues of the juvenile Yellow River carp with feed levels ranging from 25% to 34% protein content, indicating that during the process of gradually increasing dietary protein content, the *Rhag*, *Rhbg*, and *Rhcg1* genes may promote the emission of ammonia to stabilize the body environment. The lowest relative *Rhag*, *Rhbg*, and *Rhcg1* expression levels were observed with a 250 g/kg protein diet in the Yellow River carp (160.24 ± 15.56 g) [8], consistent with this study’s results. The results of this study revealed that the relative expression levels of *Rhag*, *Rhbg*, and *Rhcg1* decreased significantly when the dietary protein level was 37%. In zebrafish (*Danio rerio*), Rh family genes did not participate in promoting ammonia entry of ammonia into gill neuroepithelial cells at high ammonia levels [51,52], which is consistent with the results of this study.

In summary, this study revealed that the level of protein in the diet has significant effects on growth, muscle quality, serum biochemical indicators, the activities of digestive enzymes and antioxidant enzymes, and related functional genes in the juvenile Yellow River carp. An appropriate level of dietary protein can improve the growth, stress resistance, and protein utilization rate of common carp to a certain extent. The results of this study can provide theoretical support for the breeding of the Yellow River carp.

## 5. Conclusions

This study revealed that the optimal feed protein requirement of the juvenile Yellow River carp (51.56 ± 0.17 g) is 33.99–35.26%.

## Figures and Tables

**Table 1:** Feed formulation and nutritional levels (dry matter).

Ingredient	Dietary Protein Levels (%)					
	22	25	28	31	34	37
Fish meal %	20.00	20.00	20.00	20.00	20.00	20.00
Casein %	4.50	9.30	14.00	19.00	23.70	28.50
Fish oil %	2.50	2.50	2.50	2.50	2.50	2.50
Soybean oil %	2.50	2.50	2.50	2.50	2.50	2.50
Wheat meal %	20.00	20.00	20.00	20.00	20.00	20.00
Cornstarch %	20.00	20.00	20.00	20.00	20.00	20.00
^1^ Vitamin premix %	0.50	0.50	0.50	0.50	0.50	0.50
^2^ Mineral premix %	0.50	0.50	0.50	0.50	0.50	0.50
Choline chloride %	0.30	0.30	0.30	0.30	0.30	0.30
Ca(H_2_PO_4_)_2_ %	2.00	2.00	2.00	2.00	2.00	2.00
Cellulose %	23.70	19.60	15.50	11.30	7.30	3.20
DL-Met %	0.60	0.50	0.40	0.30	0.10	0.00
L-Thr %	1.00	0.80	0.60	0.40	0.20	0.00
L-Lys %	1.90	1.50	1.20	0.70	0.30	0.00
Total	100.00	100.00	100.00	100.00	100.00	100.00
^3^ Nutrient level %						
Crude protein %	22.63	25.32	28.15	31.43	34.18	37.27
Crude lipid %	7.00	7.10	6.80	7.10	7.00	7.20
Lys %	2.27	2.74	2.77	2.68	2.67	2.64
Met %	0.81	0.89	0.82	0.91	0.82	0.90
Thr %	1.53	1.50	1.50	1.51	1.50	1.50
Phosphorus %	1.13	1.00	0.97	0.95	1.15	0.98

Notes: ^1^ Vitamin premix (mg/kg feed): Vitamin A 36, Vitamin D_3_ 1, Vitamin E 1400, Vitamin K_3_ 350, Vitamin B_1_ 200, Vitamin B_2_ 200, Vitamin B_6_ 270, Vitamin B_12_ 1.2, Vitamin C 3500, Calcium D pantothenate 850, Nicotinamide 1000, Folic acid 85 mg, D-biotin 4.0, and Inositol 1400. ^2^ Mineral premix (mg/kg feed): Magnesium 850, Zinc 700, Manganese 370, Copper 136, Iron 3100, Cobalt 33, Iodine 20, and Selenium 10. ^3^ Nutrient levels are measured values.

**Table 2:** Effects of different dietary protein contents on growth performance of the Yellow River carp.

Growth Index	Dietary Protein Levels (%)					
	22	25	28	31	34	37
FCE(¥/kg)	33.71	44.97	56.20	67.73	78.71	90.22
IBW (g)	51.98 ± 0.47	51.04 ± 0.47	51.32 ± 0.19	51.50 ± 0.75	51.90 ± 0.85	51.16 ± 0.08
FBW (g)	148.54 ± 0.49 ^e^	162.30 ± 1.15 ^d^	171.45 ± 1.14 ^cd^	181.87 ± 0.75 ^c^	205.13 ± 2.13 ^a^	178.36 ± 1.16 ^b^
WGR (%)	185.82 ± 2.07 ^e^	218.03 ± 0.82 ^d^	234.08 ± 1.73 ^c^	253.31 ± 5.38 ^b^	295.34 ± 2.94 ^a^	248.65 ± 2.15 ^b^
SGR (%/d)	1.88 ± 0.01 ^d^	2.07 ± 0.00 ^c^	2.15 ± 0.01 ^c^	2.25 ± 0.03 ^b^	2.45 ± 0.01 ^a^	2.23 ± 0.01 ^b^
AGR (g/d)	1.74 ± 0.14 ^c^	1.87 ± 0.09 ^c^	2.13 ± 0.10 ^bc^	2.06 ± 0.27 ^bc^	2.42 ± 0.40 ^ab^	2.84 ± 0.31 ^a^
AF (g/d)	0.29 ± 0.02 ^b^	0.31 ± 0.01 ^b^	0.31 ± 0.01 ^b^	0.31 ± 0.01 ^b^	0.35 ± 0.01 ^a^	0.36 ± 0.01 ^a^
PI (g/d%)	0.06 ± 0.01 ^f^	0.09 ± 0.00 ^e^	0.09 ± 0.00 ^d^	0.10 ± 0.00 ^c^	0.12 ± 0.00 ^b^	0.13 ± 0.00 ^a^
FER	0.49 ± 0.04 ^c^	0.58 ± 0.03 ^b^	0.60 ± 0.03 ^b^	0.62 ± 0.02 ^ab^	0.67 ± 0.02 ^a^	0.60 ± 0.02 ^b^
PER (%)	1.71 ± 0.01 ^e^	1.76 ± 0.00 ^d^	1.86 ± 0.02 ^d^	1.89 ± 0.02 ^b^	2.00 ± 0.01 ^a^	1.89 ± 0.03 ^c^
FCR	2.05 ± 0.02 ^a^	1.96 ± 0.01 ^b^	1.77 ± 0.02 ^c^	1.70 ± 0.02 ^d^	1.54 ± 0.01 ^e^	1.70 ± 0.01 ^d^
SR (%)	100.00	100.00	100.00	100.00	100.00	100.00

Note: Data are presented as the mean ± SDs (*n* = 3). The values with different superscript letters within the same line are significantly different (*p* < 0.05).

**Table 3:** Effects of different dietary protein content on the amino acid content of the muscle of the Yellow River carp (g/100 g, dry weight).

Amino Acid	Dietary Protein Levels (%)					
	22	25	28	31	34	37
Asp *	1.52 ± 0.01	1.52 ± 0.01	1.51 ± 0.01	1.52 ± 0.01	1.51 ± 0.01	1.48 ± 0.00
Thr ^#^	0.65 ± 0.01 ^c^	0.66 ± 0.01 ^bc^	0.67 ± 0.01 ^ab^	0.68 ± 0.01 ^a^	0.69 ± 0.01 ^a^	0.67 ± 0.01 ^ab^
Ser ^●^	0.55 ± 0.01	0.52 ± 0.01	0.54 ± 0.01	0.53 ± 0.01	0.52 ± 0.01	0.52 ± 0.01
Glu *	2.06 ± 0.01 ^c^	2.07 ± 0.01 ^c^	2.10 ± 0.00 ^b^	2.12 ± 0.01 ^b^	2.15 ± 0.01 ^a^	2.17 ± 0.01 ^a^
Gly *	0.71 ± 0.01	0.72 ± 0.01	0.71 ± 0.01	0.70 ± 0.01	0.71 ± 0.01	0.70 ± 0.00
Ala *	0.93 ± 0.01	0.93 ± 0.01	0.92 ± 0.01	0.93 ± 0.01	0.93 ± 0.01	0.91 ± 0.00
Cys ^●^	0.13 ± 0.01 ^c^	0.14 ± 0.01 ^bc^	0.14 ± 0.01 ^abc^	0.15 ± 0.01 ^abc^	0.16 ± 0.01 ^ab^	0.16 ± 0.01 ^a^
Val ^#^	0.72 ± 0.01 ^c^	0.75 ± 0.01 ^b^	0.77 ± 0.01 ^b^	0.77 ± 0.01 ^b^	0.80 ± 0.01 ^a^	0.77 ± 0.00 ^ab^
Met ^#^	0.37 ± 0.01	0.36 ± 0.01	0.37 ± 0.01	0.37 ± 0.01	0.37 ± 0.01	0.37 ± 0.01
Ile ^#^	0.65 ± 0.00 ^c^	0.66 ± 0.00 ^c^	0.67 ± 0.01 ^bc^	0.69 ± 0.01 ^ab^	0.70 ± 0.00 ^a^	0.69 ± 0.01 ^ab^
Leu ^#^	1.22 ± 0.01	1.23 ± 0.00	1.22 ± 0.00	1.23 ± 0.00	1.24 ± 0.01	1.23 ± 0.00
Tyr ^●^	0.44 ± 0.01	0.43 ± 0.01	0.44 ± 0.01	0.43 ± 0.01	0.43 ± 0.01	0.43 ± 0.01
Phe ^#^	0.61 ± 0.01	0.62 ± 0.01	0.61 ± 0.00	0.61 ± 0.01	0.61 ± 0.01	0.61 ± 0.01
Lys ^#^	1.48 ± 0.01 ^d^	1.48 ± 0.01 ^d^	1.50 ± 0.00 ^c^	1.55 ± 0.01 ^b^	1.57 ± 0.01 ^a^	1.54 ± 0.00 ^b^
His ^※^	0.59 ± 0.01	0.59 ± 0.01	0.58 ± 0.01	0.59 ± 0.01	0.58 ± 0.01	0.57 ± 0.00
Arg ^※^	0.89 ± 0.01	0.90 ± 0.00	0.89 ± 0.00	0.89 ± 0.01	0.89 ± 0.01	0.88 ± 0.01
Pro ^●^	0.51 ± 0.00	0.50 ± 0.01	0.49 ± 0.01	0.49 ± 0.01	0.48 ± 0.01	0.48 ± 0.01
EAA	5.69 ± 0.02 ^e^	5.75 ± 0.02 ^d^	5.81 ± 0.01 ^c^	5.89 ± 0.01 ^b^	5.98 ± 0.01 ^a^	5.87 ± 0.01 ^b^
HEAA	1.48 ± 0.01	1.49 ± 0.00	1.48 ± 0.01	1.48 ± 0.00	1.46 ± 0.01	1.45 ± 0.01
FAA	5.22 ± 0.00 ^d^	5.23 ± 0.01 ^cd^	5.24 ± 0.01 ^bcd^	5.28 ± 0.01 ^ab^	5.29 ± 0.01 ^a^	5.26 ± 0.01 ^abc^
NEAA	8.31 ± 0.01	8.31 ± 0.02	8.33 ± 0.02	8.35 ± 0.01	8.35 ± 0.00	8.31 ± 0.02
TAA	14.01 ± 0.01 ^d^	14.07 ± 0.01 ^d^	14.14 ± 0.02 ^c^	14.24 ± 0.01 ^b^	14.33 ± 0.01 ^a^	14.18 ± 0.03 ^bc^
EAA/TAA %	40.65 ± 0.12 ^d^	40.85 ± 0.11 ^cd^	41.10 ± 0.06 ^bc^	41.39 ± 0.02 ^b^	41.66 ± 0.09 ^a^	41.41 ± 0.09 ^b^
NEAA/TAA %	59.35 ± 0.12 ^a^	59.10 ± 0.11 ^ab^	58.90 ± 0.06 ^b^	58.59 ± 0.02 ^c^	58.27 ± 0.05 ^d^	58.18 ± 0.03 ^d^
HEAA/TAA %	10.57 ± 0.11 ^a^	10.59 ± 0.03 ^a^	10.44 ± 0.08 ^ab^	10.40 ± 0.01 ^ab^	10.21 ± 0.07 ^bc^	10.11 ± 0.03 ^c^
FAA/TAA %	37.24 ± 0.03 ^a^	37.20 ± 0.10 ^ab^	37.07 ± 0.07 ^ab^	37.06 ± 0.04 ^ab^	36.94 ± 0.10 ^b^	37.09 ± 0.05 ^ab^
Nutrient Composition (%)						
Moisture	76.30 ± 0.05	76.20 ± 0.05	76.27 ± 0.07	76.20 ± 0.05	76.13 ± 0.11	76.23 ± 0.03
CP	19.07 ± 0.08 ^d^	19.20 ± 0.05 ^d^	19.43 ± 0.03 ^c^	19.70 ± 0.08 ^b^	19.96 ± 0.07 ^a^	19.60 ± 0.05 ^bc^
CL	2.07 ± 0.05 ^a^	1.97 ± 0.03 ^ab^	1.97 ± 0.03 ^ab^	1.93 ± 0.03 ^ab^	1.87 ± 0.03 ^b^	1.93 ± 0.03 ^ab^
Ash	1.07 ± 0.03	1.07 ± 0.03	1.07 ± 0.03	1.07 ± 0.03	1.07 ± 0.03	1.03 ± 0.03
Total sugar	0.53 ± 0.01	0.54 ± 0.00	0.55 ± 0.02	0.57 ± 0.01	0.55 ± 0.00	0.54 ± 0.00

Note: * represents flavor amino acids (FAA), # represents essential amino acids (EAA), ※ represents half essential amino acids (HEAA), and ● is nonessential amino acid (NEAA). Data are presented as the mean ± SDs (*n* = 9). The values with different superscript letters within the same line are significantly different (*p* < 0.05).

**Table 4:** Effects of different dietary protein contents on the fatty acids of the muscle of the Yellow River carp (g/100 g, dry weight).

Fatty Acid	Dietary Protein Levels (%)					
	22	25	28	31	34	37
C14:0	0.032 ± 0.001 ^b^	0.031 ± 0.001 ^b^	0.036 ± 0.002 ^b^	0.033 ± 0.001 ^b^	0.036 ± 0.002 ^b^	0.042 ± 0.000 ^a^
C15:0	0.005 ± 0.000	0.005 ± 0.000	0.005 ± 0.000	0.005 ± 0.000	0.006 ± 0.000	0.005 ± 0.000
C16:0	0.383 ± 0.003	0.385 ± 0.004	0.373 ± 0.014	0.374 ± 0.013	0.368 ± 0.008	0.400 ± 0.002
C18:0	0.112 ± 0.002 ^ab^	0.107 ± 0.003 ^b^	0.117 ± 0.003 ^a^	0.108 ± 0.002 ^b^	0.107 ± 0.002 ^b^	0.119 ± 0.002 ^a^
SFA	0.532 ± 0.001 ^b^	0.528 ± 0.007 ^b^	0.531 ± 0.012 ^b^	0.520 ± 0.014 ^b^	0.516 ± 0.009 ^b^	0.567 ± 0.004 ^a^
C16:1	0.067 ± 0.005	0.063 ± 0.009	0.073 ± 0.006	0.075 ± 0.005	0.077 ± 0.003	0.079 ± 0.004
C18:1n9c	0.486 ± 0.019	0.561 ± 0.043	0.543 ± 0.051	0.559 ± 0.030	0.570 ± 0.045	0.587 ± 0.019
C20:1	0.038 ± 0.003	0.041 ± 0.003	0.043 ± 0.004	0.049 ± 0.003	0.045 ± 0.001	0.045 ± 0.001
MUFA	0.606 ± 0.024	0.678 ± 0.050	0.675 ± 0.060	0.698 ± 0.038	0.706 ± 0.048	0.726 ± 0.017
C22:1n9	0.008 ± 0.000 ^a^	0.006 ± 0.000 ^b^	0.007 ± 0.000 ^a^	0.007 ± 0.000 ^ab^	0.007 ± 0.001 ^ab^	0.008 ± 0.000 ^a^
C24:1	0.008 ± 0.000	0.007 ± 0.000	0.008 ± 0.000	0.008 ± 0.000	0.008 ± 0.000	0.008 ± 0.000
C18:2n6c	0.202 ± 0.006 ^b^	0.231 ± 0.003 ^a^	0.224 ± 0.007 ^ab^	0.232 ± 0.004 ^a^	0.243 ± 0.002 ^ab^	0.225 ± 0.009 ^ab^
C18:3n3	0.018 ± 0.001	0.020 ± 0.001	0.017 ± 0.001	0.021 ± 0.002	0.021 ± 0.001	0.021 ± 0.001
C20:2	0.008 ± 0.000	0.007 ± 0.000	0.008 ± 0.000	0.008 ± 0.000	0.007 ± 0.000	0.008 ± 0.000
C20:3n6	0.017 ± 0.001 ^b^	0.014 ± 0.000 ^c^	0.016 ± 0.000 ^b^	0.014 ± 0.000 ^c^	0.017 ± 0.000 ^ab^	0.019 ± 0.000 ^a^
C20:4n6	0.044 ± 0.002	0.045 ± 0.002	0.049 ± 0.003	0.051 ± 0.001	0.045 ± 0.002	0.051 ± 0.001
C20:5n3 (EPA)	0.035 ± 0.002 ^ab^	0.040 ± 0.001 ^ab^	0.034 ± 0.001 ^b^	0.041 ± 0.002 ^a^	0.041 ± 0.002 ^a^	0.040 ± 0.001 ^a^
C22:6n3 (DHA)	0.131 ± 0.001	0.132 ± 0.004	0.140 ± 0.002	0.140 ± 0.001	0.140 ± 0.001	0.137 ± 0.004
PUFA	0.456 ± 0.007 ^b^	0.488 ± 0.008 ^a^	0.487 ± 0.002 ^a^	0.507 ± 0.006 ^a^	0.514 ± 0.003 ^a^	0.500 ± 0.012 ^a^
DHA+EPA	0.1667 ± 0.003 ^b^	0.172 ± 0.003 ^ab^	0.174 ± 0.003 ^ab^	0.181 ± 0.001 ^a^	0.181 ± 0.001 ^a^	0.177 ± 0.003 ^a^
n-3 PUFA	0.185 ± 0.003 ^c^	0.192 ± 0.003 ^abc^	0.191 ± 0.002 ^bc^	0.202 ± 0.002 ^a^	0.202 ± 0.001 ^a^	0.198 ± 0.003 ^ab^
n-6 PUFA	0.263 ± 0.004 ^b^	0.289 ± 0.005 ^a^	0.289 ± 0.005 ^a^	0.297 ± 0.005 ^a^	0.304 ± 0.002 ^a^	0.294 ± 0.009 ^a^
n-3 PUFA/n-6 PUFA	0.703 ± 0.001	0.663 ± 0.003	0.662 ± 0.020	0.679 ± 0.009	0.664 ± 0.006	0.674 ± 0.009
TFA	1.594 ± 0.025	1.694 ± 0.063	1.693 ± 0.051	1.725 ± 0.058	1.736 ± 0.0057	1.794 ± 0.020

Note: Data are presented as the mean ± SDs (*n* = 9). The values with different superscript letters within the same line are significantly different (*p* < 0.05).

**Table 5:** Effects of different dietary protein contents on serum biochemical indicators of the Yellow River carp.

Biochemical Index	Dietary Protein Levels (%)					
	22	25	28	31	34	37
ALP (U·L^−1^)	21.10 ± 0.42 ^d^	27.37 ± 0.28 ^c^	30.47 ± 0.30 ^c^	37.77 ± 2.85 ^b^	43.20 ± 0.99 ^a^	35.63 ± 0.84 ^b^
TP (g·L^−1^)	33.73 ± 0.73	34.03 ± 0.1	32.13 ± 1.8	32.53 ± 0.31	33.87 ± 0.22	32.50 ± 0.71
UA (μmol·L^−1^)	20.90 ± 0.08 ^c^	32.90 ± 1.24 ^b^	37.57 ± 2.05 ^ab^	38.57 ± 1.88 ^a^	41.00 ± 1.97 ^a^	41.17 ± 0.91 ^a^

Note: Data are presented as the mean ± SDs (*n* = 3). The values with different superscript letters within the same line are significantly different (*p* < 0.05).

## Data Availability

Data will be made available upon request.

## Supplementary Materials

The following supporting information can be downloaded at https://www.mdpi.com/article/10.3390/ani15121800/s1. Table S1: All primer sequences in this study.

## Abbreviations

The following abbreviations are used in this manuscript:

SR	Survival rate
WGR	Weight gain rate
SGR	Specific growth rate
FCR	Feed conversion ratio
PER	Protein efficiency ratio
TP	Total protein
GSH-Px	Glutathione peroxidase enzyme
CAT	Catalase
MDA	Malondialdehyde
TBA	Thiobarbituric acid
SOD	Superoxide dismutase
α-AMS	α-amylase
LPS	Lipase
TPS	Trypsin
ALP	Phosphatase
UA	Uric acid
β-actin	Beta-actin
GH	Growth hormone
*IGF-I*	Insulin-like growth factor I
TOR	Rapamycin target protein
*4EBP2*	Eukaryotic translation initiation factor 4e binding protein 2
*Rhag*	Rhesus-associated glycoprotein
*Rhbg*	Rhesus type B glycoprotein
*Rhcg1*	Rh family C glycoprotein 1
ANOVA	One-way analysis of variance
FCE	Feed cost estimate
AF	Absolute feed of each fish
PI	Protein intake of each fish
FER	Feed efficiency ratio
FBW	Final body weight
IBW	Initial body weight
CP	Crude protein
CL	Crude lipid
Asp	Aspargine
Thr	Threonine
Ser	Serine
Glu	Glutamine
Gly	Glycine
Ala	Alanine
Cys	Cystine
Val	Valine
Met	Methionine
Ile	Isoleucine
Leu	Leucine
Tyr	Tyrosine
Phe	Phenylalanine
Lys	Lysine
His	Histidine
Arg	Arginine
Pro	Proline
EAA	Essential amino acid
HEAA	Half essential amino acids
FAA	Flavor amino acid
NEAA	Non-essential amino acids
TAA	Total amino acids
SFA	Saturated fatty acid
MUFA	Monounsaturated fatty acid
EPA	Eicosapentaenoic acid
DHA	Docosahexaenoic acid
PUFA	Polyunsaturated fatty acid
TFA	Total fatty acid
