# Supplementary material for: Effects of Dietary Protein Levels on the Growth, Physiological, and Biochemical Indices of Juvenile Yellow River Carp (Cyprinus carpio haematopterus)
Source: Animals (Basel). 2025 Jun 18;15(12):1800. doi: 10.3390/ani15121800 (PMC12189580; doi:10.3390/ani15121800)
Supplement: Supplementary file 1 [file animals-15-01800-s001.zip › animals-3655050-supplementary.pdf]

Supplementary Table S1. All primers sequences in this study

Primer	Sequence (5'-3')	Tm	Application
GH	F: GGGGAGAGCATCAGACAACC<br>R: CAGGCTGTCCTCAAAGTCGT	60℃	qRT-PCR
IGF-1	F: GGCGCCTCGAGATGTATTGT<br>R: TCCTTGGGCTGTCTGTATGC	60℃	qRT-PCR
TOR	F: AGCTAAGCCAAGATGAAGCCA<br>R: ACTATGGCCAGGATACCACCT	60℃	qRT-PCR
4EBP2	F: CCTCACGACTATTGCACCACT<br>R: CTGGGCGATGGGTGAGTTA	60℃	qRT-PCR
Rhag	F: AAAGAAAGACAATGTTACGGCCA<br>R: AATCAGGCCTGTGATCAACCC	60℃	qRT-PCR
Rhbg	F: TCGCAGCCTTTTCCCTACAG<br>R: CTAGTCACGCCAACGTGGAT	60℃	qRT-PCR
Rhcg1	F: GTTCCTCCAGTCCTGGCTTAT<br>R: ACGCTGCTCCACGAAGTTT	60℃	qRT-PCR
β-actin	F: GCCGTGACCTGACTGACTACCT<br>R: GCCACATAGCAGAGCTTCTCCTTG	60℃	qRT-PCR
